# Psychological Impact in Healthcare Workers During Emergencies: The Italian Experience With COVID-19 First Wave

**DOI:** 10.3389/fpsyt.2022.818674

**Published:** 2022-03-21

**Authors:** Elisabetta Pisanu, Annalisa Di Benedetto, Maria Rita Infurna, Raffaella I. Rumiati

**Affiliations:** ^1^Neuroscience Area, International School for Advanced Studies (SISSA), Trieste, Italy; ^2^ANVUR, Rome, Italy; ^3^Scuola Superiore di Studi Avanzati Sapienza (SSAS), Rome, Italy

**Keywords:** COVID-19, pandemic, psychological impact, mental health, healthcare workers (HCWs)

## Abstract

**Background:**

The COVID-19 outbreak imposed an overwhelming workload as well as emotional burdens on Healthcare workers (HCWs). In May 2020, an online survey was administered to HCWs in Italy to assess the pandemic's psychological impact and to investigate possible predictive factors that led to individual differences.

**Methods:**

The psychological experience was measured based on the prevalence of self-reported feelings during the pandemic, including negative and positive emotional states. We analyzed the relationship between factors of gender, age, geographic region, professional role, and operational unit, and the four-point scale used to rate the frequency of each emotional state experienced by performing several multinomial logistic regressions, one for each emotion.

**Results:**

Our findings suggest that more than half of HCWs experienced psychological distress during the first COVID-19 outbreak in Italy. Female and younger respondents, especially those operating in northern Italy experienced more frequently negative emotional states such as irritability, anxiety, loneliness, and insecurity. However, positive feelings, first of all solidarity, were also reported especially by female and older workers. The majority of the negative as well as positive emotional states were experienced almost equally by both doctors and nurses, and independently of the operational unit in which they operated.

**Conclusions:**

This study can be very useful as a contribution to the current literature on the psychological effects of this pandemic on health workers. Moreover, our findings can provide useful information in planning more tailored psychological interventions to support this category of workers in the ongoing and future emergencies.

## Introduction

On December 31, 2019, the World Health Organization (WHO) received the news about an unusual rise in pneumonia cases in the city of Wuhan, China. This was the first manifestation of the coronavirus disease-19 (COVID-19) caused by an acute and highly contagious virus (SARS-CoV-2) that rapidly affects the respiratory system (1). Due to the rapid increase in the number of cases outside of China, on March 11, 2020, the WHO general director announced the global pandemic (2), leading to a global health emergency that has strongly marked and affected our era.

In Italy, the first outbreak of COVID-19 began at the end of February 2020 in the North and then rapidly spread to the rest of the country. Consequently, in order to limit the infection, the Government declared the *lockdown* from March 9 until May 3 of the same year. During this first wave, highly restrictive measures were adopted such as physical and social distancing, quarantine, movement restrictions, military control (3). In the following summer, given the reduction in the number of infections, the restrictions were revised with the re-opening of commercial activities after adopting safe measures ensuring social distancing and specific hygiene rules to avoid the contagion. However, with new waves of infections, from the end of October 2020 new restrictions were adopted, including the closure of numerous activities (schools, restaurants, bars, gyms, swimming pools, cinemas, theaters etc.), movement limitations, and the introduction of the curfew (from 10 pm to 5 am) (4). Furthermore, color coded zones were established throughout Italy defined by specific parameters to be adopted individually by each region, based on the level of risk of the virus spreading (*Rt* index). In May 2021, we exited what was defined as the third wave thanks to a successful vaccine campaign which has clearly helped keeping the spread of the virus under control. However, at the end of July, we have entered the fourth wave fuelled by the delta variant of the virus.

This pandemic can be defined as one of the most challenging of the twenty-first century for the scientific communities and societies world-wise (5). The socio-economic impact of the COVID-19 pandemic is upsetting, characterized by a global economic loss due to the abovementioned measures adopted to contain the spread of the virus (6–8).

Although the economic aspect is pivotal, the severe impact on the population's mental health is no less important (9–13). Indeed, we can refer to this situation as a collective trauma, during which we have been living our daily life in a dramatic climate of uncertainty, fear and loss (14, 15). The fear of contracting the virus, as well as the fear of infecting other members of the family, in a climate of total loss of control where social relationships are discouraged, has led to a strong increase of mental diseases such as anxiety and depression (9, 16–18). Furthermore, repeated media exposure as well as the spread of fake or contradictory news has heightened stress responses, negatively affecting health overall (19, 20).

The COVID-19 outbreak has imposed an overwhelming workload as well as emotional burdens in particular on Healthcare workers (HCWs). Indeed, since the beginning of the health emergency, they have been on the frontline fighting the epidemic, being at higher risk of becoming infected and experiencing an emotional overload. The literature on work-related stress has reported the presence of psychosocial risk factors in the healthcare sector (21–23) that are associated with staff's working conditions, safety and health: the emergency has been amplifying these factors (23–26). Psychological and physical stress among HCWs could be also increased by social isolation, social distancing and quarantine measures or even discrimination as potentially infected people in the common imagination, and the lack of family support due to fear of infection (27–29). Furthermore, the psychological distress might have been enhanced by the lack of effective treatments and shortages of dedicated equipment, as well as by witnessing people dying alone, without their loved ones (30, 31).

As a result, HCWs might have felt angry, hostile, frustrated or helpless, experience symptoms of depression and anxiety accompanied by physical complaints, and suffer from insomnia (25, 32–34). Additionally, frontline HCWs are also exposed to the risk of developing secondary stress disorder by taking care of patients who are both physically and psychologically suffering from the emergency (32, 35). Because of this strong physical and emotional overload experienced by HCWs, various listening and psychological support numbers as well as teleconsultation services have been activated. However, only a small number of them exploited these services and their effectiveness still remains unclear (36–38). Recent studies reported that sometimes these services were not considered adequate enough by HCWs because they are disorganized, difficult to reach, incompatible with HCWs' work schedules, with an insufficient number of sessions, and characterized by an individual modality (typically, *ad hoc* created listening services). In contrast, a group approach would have been more adequate as it allows sharing needs and difficulties together. However, HCWs also reported to believe their problems were not severe enough to require these services and to be able to manage them on their own, despite the high psychological distress reported (39–42).

The main aims of this study consist in analyzing the nature and the severity of the mental complaints reported by the HCWs during the first COVID-19 outbreak in Italy, and in highlighting possible predictive factors that led to significant differences in experiencing this psychological distress. A further aim is to analyze the possible experience of positive emotions, in spite of the dramatic situation, to highlight possible protective factors. In fact, positive emotions have been associated with increased well being and improved psychological resources needed for adaptive coping (43, 44).

## Materials and Methods

### Study Design and Sampling

Two *ad hoc* questionnaires were designed to be administered online via Google Forms specifically to doctors and other healthcare workers. Respondents were invited to participate in the study via social media (Facebook, Whatsapp) and email, as well as through the website of scientific societies. The procedure involved filling in an online consent form and all data were collected anonymously and organized in electronic format in the password-protected Google Drive archive. The questionnaires were answered individually and voluntarily by participants. The survey was run from April 28 to May 31 2020. The study and procedures of informed consent have been approved by the corresponding author's institutional ethics committee.

### Measures

Two structured questionnaires were designed and administered to HCWs operating in Italy. Both questionnaires consisted of 31 closed-ended questions dedicated to their emotional experience during the emergency. Moreover, beyond the demographic information including age, gender, geographical place of employment, professional role, and operational unit, different thematic areas were addressed:

- Possible sources of work-related stress (temporal and content aspects of the workday and the work activity, the organization conditions);
- Specific aspects related to COVID-19 (emotional responses, stress factors specific to frontline staff, resilience and psychosocial support);
- Governance and care responsibilities (governance actions and medical support, psychological actions and tools adopted, psychological assessment areas).

In this study we analyzed in particular the psychological impact. This was measured based on the prevalence of self-reported feelings during the pandemic, including negative and positive emotional states, such as loneliness, anxiety, irritability, sadness, tiredness, insecurity, apathy, intolerance, frustration, insomnia, fear, impatience, impotence, anger, resignation, pride, satisfaction, trust, hope, solidarity, quiet (“*During the emergency, how often did you feel..*.”). The responses were scored on a four-point Likert scale, depending on the frequency of each feeling experienced (“*Never or almost never,”* “*Sometimes*,” “*Often*,” “*Always or almost always*”).

### Data Analysis

Descriptive statistics were carried out to analyse categorical variables; percentage of responses was calculated according to the number of respondents for each question compared to the total number of responses to a question.

We analyzed the relationship between factors of gender, age, geographic region, professional role, and operational unit, and the four-point scale used to rate the frequency of each emotional state experienced by performing several multinomial logistic regressions, one for each emotion, using the R function “multinom” (45). We performed this analysis to test whether the five abovementioned factors could be good predictors of the emotional experience by considering each emotion independently. Therefore, we built several models, one for each emotion that represented our categorical dependent variable with four levels, where we entered the five factors as independent categorical variables. The categorical nature of our variables made suitable this type of analysis; however, the data were previously evaluated to ensure that all the other model's assumptions were fulfilled too (sample size, outliers, multicollinearity). More specifically, first we used G^*^Power (46) software to confirm the minimum sample size necessary to detect a small population effect size at power = 0.95 for α = 0.05 for the study's number of variables. Then, we checked carefully our data to avoid the possibility of outliers, and we ruled out multicollinearity by means of a correlation matrix.

Additionally, Spearman rank correlation was computed to assess correlations with all the emotions. *P* values < 0.05 were considered statistically significant, and missing values were excluded for analysis purposes. Data were processed and analyzed in accordance with the privacy protection legislation, and the results of the data analysis were disclosed exclusively in aggregate form.

Furthermore, we performed the Harman's single factor test by using the R function “fa” and choosing the principal axis factoring for extraction to rule out common-method variance bias.

## Results

### Sample Details

In total 577 people completed the online survey. One participant was excluded due to an excessive lack of demographic information, yielding a final sample of 576 participants (68% females) with mean age of 44.3 (SD = 11.9, range = 22–69). Of these, 38.7% were doctors, while 61.3% were other Healthcare workers, mostly nurses (81%) and for this reason, in the tables and in the results section, we used the label “nurses” to indicate the respondents belonging to all the other healthcare professions involved. About 68.9% of the sample was from northern Italy (54.5% North-East, 14.4% North-West), and 30.9% was from central-southern regions (21% central regions, 8.3% South, 1.6% islands). Regarding the operating unit or department, 16.3% worked within the *ad hoc* created COVID units, 5.6% in anesthesia, reanimation and intensive care, and 73.4% in other departments. Table 1 summarizes the details of the study sample.

**Table 1:** Demographic characteristics of the sample.

***N*** **= 576**	***N*** **(%)**
**Gender**	
F	392 (68.1%)
M	184 (31.9%)
**Age category**	
≤ 34	162 (28.1%)
35–54	268 (46.5%)
≥55	146 (25.3%)
**Professional role**	
Doctor	223 (38.7%)
Nurse	353 (61.3%)
**Region of Italy**	
North	397 (68.9%)
Centre-South	178 (30.9%)
Missing	1 (0.2%)
**Operational Unit**	
Anesthesia/Reanimation/Intensive care	32 (5.6%)
New COVID unit	94 (16.3%)
Other	423 (73.4%)
Missing	27 (4.7%)

### Psychological Impact

Descriptive analysis showed that more than half of the HCWs experienced all the emotional states investigated, in respecting of the valance, with the exception of apathy (30.5%), at least sometimes (loneliness 54.2%, anxiety 76.5%, irritability 81.2, sadness 75%, tiredness 87.2%, insecurity 72.3%, intolerance 64.4%, frustration 67.2%, insomnia 57.3%, fear 63.3%, impatience 59.5%, impotence 72.8%, anger 67.8%, resignation 51.5%, pride 68.6%, satisfaction 83.8%, trust 87.5%, hope 90.4%, solidarity 94.8%, quiet 79.9%) (Tables 2, 3). Correlation analysis across all the emotional states experience during the COVID-19 outbreak is reported in Table 4.

**Table 2:** Self-reported prevalence of negative feelings.

***N*** **= 576**	***N*** **(%)**	***N*** **= 576**	***N*** **(%)**	***N*** **= 576**	***N*** **(%)**
**Loneliness**		**Insecurity**		**Fear**	
Never or almost never	264 (45.8%)	Never or almost never	160 (27.8%)	Never or almost never	212 (36.8%)
Sometimes	213 (37.0%)	Sometimes	278 (48.3%)	Sometimes	266 (46.2%)
Often	89 (15.5%)	Often	123 (21.4%)	Often	85 (14.8%)
Almost always or always	10 (1.7%)	Almost always or always	15 (2.6%)	Almost always or always	13 (2.3%)
**Anxiety**		**Intolerance**		**Impatience**	
Never or almost never	135 (23.4%)	Never or almost never	205 (35.6%)	Never or almost never	233 (40.5%)
Sometimes	276 (47.9%)	Sometimes	238 (41.3%)	Sometimes	242 (42.0%)
Often	139 (24.1%)	Often	118 (20.5%)	Often	87 (15.1%)
Almost always or always	26 (4.5%)	Almost always or always	15 (2.6%)	Almost always or always	14 (2.4%)
**Irritability**		**Frustration**		**Impotence**	
Never or almost never	106 (18.4%)	Never or almost never	189 (32.8%)	Never or almost never	157 (27.3%)
Sometimes	256 (44.4%)	Sometimes	221 (38.4%)	Sometimes	244 (42.4%)
Often	187 (32.5%)	Often	144 (25.0%)	Often	143 (24.8%)
Almost always or always	25 (4.3%)	Almost always or always	22 (3.8%)	Almost always or always	32 (5.6%)
Missing	2 (0.3%)				
**Sadness**		**Insomnia**		**Anger**	
Never or almost never	144 (25.0%)	Never or almost never	246 (42.7%)	Never or almost never	186 (32.3%)
Sometimes	263 (45.7%)	Sometimes	170 (29.5%)	Sometimes	236 (41.0%)
Often	147 (25.5%)	Often	117 (20.3%)	Often	134 (23.3%)
Almost always or always	22 (3.8%)	Almost always or always	43 (7.5%)	Almost always or always	20 (3.5%)
**Tiredness**		**Apathy**		**Resignation**	
Never or almost never	72 (12.5%)	Never or almost never	400 (69.4%)	Never or almost never	279 (48.4%)
Sometimes	232 (40.3%)	Sometimes	124 (21.5%)	Sometimes	195 (33.9%)
Often	233 (40.5%)	Often	41 (7.1%)	Often	88 (15.3%)
Almost always or always	37 (6.4%)	Almost always or always	11 (1.9%)	Almost always or always	13 (2.3%)
Missing	2 (0.3%)			Missing	1 (0.2%)

**Table 3:** Self-reported prevalence of positive feelings.

***N*** **= 576**	***N*** **(%)**	***N*** **= 576**	***N*** **(%)**
**Pride**		**Hope**	
Never or almost never	181 (31.4%)	Never or almost never	55 (9.5%)
Sometimes	216 (37.5%)	Sometimes	201 (34.9%)
Often	144 (25.0%)	Often	249 (43.2%)
Almost always or always	35 (6.1%)	Almost always or always	71 (12.3%)
**Satisfaction**		**Solidarity**	
Never or almost never	93 (16.1%)	Never or almost never	30 (5.2%)
Sometimes	272 (47.2%)	Sometimes	133 (23.1%)
Often	186 (32.3%)	Often	299 (51.9%)
Almost always or always	25 (4.3%)	Almost always or always	114 (19.8%)
**Trust**		**Quiet**	
Never or almost never	72 (12.5%)	Never or almost never	116 (20.1%)
Sometimes	266 (46.2%)	Sometimes	263 (45.7%)
Often	202 (35.1%)	Often	170 (29.5%)
Almost always or always	36 (6.2%)	Almost always or always	27 (4.7%)

**Table 4:** Spearman correlation coefficient rho: associations across all the emotional states.

	**Loneliness**	**Pride**	**Anxiety**	**Satisfaction**	**Irritability**	**Sadness**	**Tiredness**	**Insecurity**	**Intolerance**	**Trust**	**Frustration**	**Insomnia**	**Hope**	**Apathy**	**Fear**	**Solidarity**	**Impatience**	**Quiet**	**Impotence**	**Anger**
Loneliness	–																			
Pride	−0.016	–																		
Anxiety	0.331^***^	–0.099^*^	–																	
Satisfaction	–0.125^**^	0.590^***^	–0.149^***^	–																
Irritability	0.276^***^	–0.086^*^	0.520^***^	–0.151^***^	–															
Sadness	0.411^***^	−0.071	0.455^***^	–0.131^**^	0.410^***^	–														
Tiredness	0.281^***^	0.063	0.418^***^	0.003	0.458^***^	0.432^***^	–													
Insecurity	0.221^***^	−0.069	0.476^***^	–0.134^**^	0.347^***^	0.394^***^	0.338^***^	–												
Intolerance	0.300^***^	−0.078	0.353^***^	–0.259^***^	0.467^***^	0.383^***^	0.325^***^	0.416^***^	–											
Trust	–0.190^***^	0.288^***^	–0.194^***^	0.509^***^	–0.236^***^	–0.182^***^	−0.065	–0.243^***^	–0.250^***^	–										
Frustration	0.336^***^	–0.113^**^	0.461^***^	–0.253^***^	0.454^***^	0.440^***^	0.359^***^	0.378^***^	0.443^***^	–0.308^***^	–									
Insomnia	0.333^***^	0.013	0.393^***^	−0.01	0.348^***^	0.401^***^	0.400^***^	0.281^***^	0.295^***^	–0.083^*^	0.354^***^	–								
Hope	–0.130^**^	0.249^***^	–0.112^**^	0.413^***^	–0.144^***^	–0.082^*^	0.012	–0.126^**^	–0.148^***^	0.605^***^	–0.152^***^	−0.025	–							
Apathy	0.238^***^	–0.116^**^	0.293^***^	–0.180^***^	0.303^***^	0.304^***^	0.227^***^	0.297^***^	0.367^***^	–0.231^***^	0.349^***^	0.223^***^	–0.210^***^	–						
Fear	0.294^***^	−0.001	0.533^***^	−0.032	0.329^***^	0.391^***^	0.238^***^	0.416^***^	0.253^***^	–0.107^*^	0.346^***^	0.300^***^	0.003	0.250^***^	–					
Solidarity	–**0.022**	0.280^***^	0.029	0.392^***^	−0.025	0.066	0.101^*^	−0.006	–0.103^*^	0.401^***^	−0.008	0.128^**^	0.520^***^	–0.169^***^	0.092^*^	–				
Impatience	0.218^***^	−0.021	0.282^***^	–0.100^*^	0.461^***^	0.290^***^	0.294^***^	0.285^***^	0.485^***^	–0.113^**^	0.315^***^	0.298^***^	−0.072	0.428^***^	0.184^***^	−0.053	–			
Quiet	–0.253^***^	0.118^**^	–0.374^***^	0.313^***^	–0.356^***^	–0.276^***^	–0.239^***^	–0.322^***^	–0.240^***^	0.502^***^	–0.293^***^	–0.208^***^	0.421^***^	–0.156^***^	–0.272^***^	0.242^***^	–0.195^***^	–		
Impotence	0.275^***^	−0.053	0.395^***^	–0.193^***^	0.324^***^	0.450^***^	0.266^***^	0.350^***^	0.359^***^	–0.174^***^	0.492^***^	0.351^***^	−0.028	0.279^***^	0.380^***^	0.151^***^	0.326^***^	–0.251^***^	–	
Anger	0.286^***^	−0.059	0.362^***^	–0.139^***^	0.490^***^	0.464^***^	0.313^***^	0.301^***^	0.415^***^	–0.202^***^	0.462^***^	0.288^***^	–0.094^*^	0.348^***^	0.304^***^	−0.026	0.411^***^	–0.254^***^	0.492^***^	–
Resignation	0.274^***^	–0.083^*^	0.266^***^	–0.195^***^	0.248^***^	0.327^***^	0.232^***^	0.313^***^	0.390^***^	–0.181^***^	0.352^***^	0.198^***^	–0.166^***^	0.385^***^	0.245^***^	–0.108^**^	0.331^***^	–0.197^***^	0.423^***^	0.427^***^

*Bold data indicate significant correlations;*

*** *p < 0001,*

** *p < 0.01,*

* *p < 0.05*.

Multinomial logistic regressions determined the relationship between demographic factors of gender, age, geographic region, professional role, and operational unit and scores (never, sometimes, often, always) obtained from the psychological impact category (loneliness, anxiety, irritability, sadness, tiredness, insecurity, apathy, intolerance, frustration, insomnia, fear, impatience, impotence, anger, resignation, pride, satisfaction, trust, hope, solidarity, quiet) (Tables 5, 6).

**Table 5:** Multinomial logistic regressions omnibus Likelihood Ratio tests for psychological impact category encompassing negative feelings and demographic factors (gender, age, geographical region, professional role, operational unit).

	**χ^2^**	**Df**	***p***
**Gender**			
Loneliness	7.94	3	**0.047**
Anxiety	24.83	3	**<0.001**
Irritability	10.57	3	**0.014**
Sadness	14.72	3	**0.002**
Tiredness	16.1	3	**0.001**
Insecurity	18.29	3	**<0.001**
Intolerance	10.41	3	**0.015**
Frustration	15.5	3	**0.016**
Insomnia	24.65	3	**<0.001**
Apathy	4.47	3	0.214
Fear	22.811	3	**<0.001**
Impatience	0.678	3	0.878
Impotence	13.14	3	**0.004**
Anger	3.96	3	0.266
Resignation	3.16	3	0.367
**Age**			
Loneliness	29.07	6	**<0.001**
Anxiety	14.93	6	**0.021**
Irritability	16.60	6	**0.011**
Sadness	7.96	6	0.240
Tiredness	9.95	6	0.126
Insecurity	21.80	6	**0.001**
Intolerance	11.41	6	0.076
Frustration	7.95	6	0.241
Insomnia	2.10	6	0.91
Apathy	15.67	6	**0.016**
Fear	5.58	6	0.472
Impatience	10.27	6	0.114
Impotence	11.14	6	0.084
Anger	7.88	6	0.247
Resignation	11.74	6	0.068
**Region**			
Loneliness	7.96	3	**0.047**
Anxiety	11.13	3	**0.011**
Irritability	10.06	3	**0.018**
Sadness	3.066	3	0.381
Tiredness	14.6	3	**0.002**
Insecurity	12.06	3	**0.007**
Intolerance	9.22	3	**0.026**
Frustration	13	3	**0.005**
Insomnia	5.8	3	0.123
Apathy	3.86	3	0.277
Fear	1.35	3	0.717
Impatience	5.76	3	0.124
Impotence	9.28	3	**0.026**
Anger	6.87	3	0.076
Resignation	4.1	3	0.251
**Professional Role**			
Loneliness	2.89	3	0.409
Anxiety	3.39	3	0.335
Irritability	4.09	3	0.252
Sadness	1.31	3	0.727
Tiredness	16.1	3	**0.001**
Insecurity	3.23	3	0.35
Intolerance	6.69	3	0.082
Frustration	3.72	3	0.293
Insomnia	1.46	3	0.69
Apathy	6.26	3	0.099
Fear	0.437	3	0.933
Impatience	10.97	3	**0.012**
Impotence	1.53	3	0.674
Anger	2.68	3	0.443
Resignation	4.42	3	0.219
**Operational unit**			
Loneliness	10.28	6	0.113
Anxiety	15.80	6	**0.015**
Irritability	8.61	6	0.197
Sadness	8.78	6	0.186
Tiredness	15.1	6	**0.019**
Insecurity	7.15	6	0.307
Intolerance	2.84	6	0.828
Frustration	1551	6	**0.016**
Insomnia	10.99	6	0.088
Apathy	4.87	6	0.559
Fear	7.86	6	0.248
Impatience	3.42	6	0.754
Impotence	4.97	6	0.548
Anger	4.8	6	0.569
Resignation	4.18	6	0.651

*Significant P values are highlighted in bold*.

*Chi-square value, degrees of freedom and significance are reported*.

**Table 6:** Multinomial logistic regressions omnibus Likelihood Ratio for psychological impact category encompassing positive feelings and demographic factors (gender, age, geographical region, professional role, operational unit).

	**χ^2^**	**Df**	***p***
**Gender**			
Quiet	23.16	3	**<0.001**
Solidarity	8.5	3	**0.036**
Hope	5.22	3	0.156
Trust	7.97	3	**0.046**
Satisfaction	15.22	3	**0.002**
Pride	2.892	3	0.409
**Age**			
Quiet	20.96	6	**0.001**
Solidarity	19.51	6	**0.003**
Hope	24.55	6	**<0.001**
Trust	18.25	6	**0.005**
Satisfaction	9.45	6	0.150
Pride	5.429	6	0.490
**Region**			
Quiet	2.48	3	0.478
Solidarity	7.07	3	0.069
Hope	2.23	3	0.526
Trust	1.43	3	0.696
Satisfaction	2.77	3	0.428
Pride	0.338	3	0.953
**Professional role**			
Quiet	6	3	0.111
Solidarity	0.43	3	0.935
Hope	1.65	3	0.648
Trust	1.62	3	0.654
Satisfaction	9.62	3	**0.022**
Pride	1.758	3	0.624
**Operational unit**			
Quiet	7.73	6	0.257
Solidarity	12.90	6	**0.044**
Hope	2.48	6	0.87
Trust	7.60	6	0.269
Satisfaction	5.49	6	0.483
Pride	6.323	6	0.388

*Significant P values are highlighted in bold*.

*Chi-square value, degrees of freedom and significance are reported*.

Furthermore, Harman's single factor test showed the total variance explained by a single factor was 28%, which falls well below the threshold of 50%. Thus, common method bias does not appear to be a significant factor in the current research.

### Multinomial Logistic Regression: Negative Feelings

*Gender* was found to be a good predictor of all negative feelings, except for apathy, impatience, anger, and resignation. These last four emotions seemed to be equally not well predicted by the gender factor; among the others, the relationship between gender and loneliness was the one with the lowest significance, while those with insecurity, insomnia, and fear showed high significance. Females experienced more distress (loneliness 58.1%, anxiety 82.1%, irritability 83.6%, sadness 79.3%, tiredness 90.4%, insecurity 72.3%, intolerance 78%, frustration 69.6%, insomnia 64.3%, fear 69.7%, impotence 76%) than males (loneliness 45.7%, anxiety 64.7%, irritability 76%, sadness 65.8%, tiredness 80.5%, insecurity 59.8%, intolerance 56.5%, frustration 62%, insomnia 42.4%, fear 49.5%, impotence 65.8%).

*Age* was predictive of loneliness, and insecurity, with a high significance, and of anxiety, irritability, and apathy with a medium significance; however it did not affect the other feelings among which, resignation was the only one to approach a low significance although without reaching it. The <34-year-old age group experienced psychological distress more often (loneliness 67.9%, anxiety 83.3%, irritability 84%, insecurity 79%, apathy 40.1%) than the > 55 year-old-age group (loneliness 43.1%, anxiety 72%, irritability 78.8%, insecurity 65.7%, apathy 25.7%).

*Region* was found to be a good predictor of loneliness, anxiety, irritability, tiredness, insecurity, intolerance, frustration, and impotence. The relationship between region and loneliness was the one with the lowest significance, while those with tiredness, insecurity, and frustration showed quite high significance. Respondents from northern Italy showed higher distress (loneliness 52.9%, anxiety 79.4%, irritability 83.3%, tiredness 89.4%, insecurity 76.8%, intolerance 67.8%, frustration 72%, and impotence 76.5%) than those working in the central-southern Italy (loneliness 46.1%, anxiety 70.3%, irritability 76.4%, tiredness 82%, insecurity 62.4%, intolerance 57.3%, frustration 56.7%, and impotence 64.7%).

With regard to the *Professional role*, only tiredness and impatience were found to be predicted by this factor, with a high significance for the former and a medium one for the latter; doctors reported feeling tired always or almost always (9.4%) and impatient often (19.7%) to a greater extent than nurses (respectively 4.5% and 12.2%). All the other feelings were far from being affected by this factor. This data shows that all health workers experienced psychological stress almost equally.

Lastly, *Operational unit* was predictive only of anxiety, tiredness, and frustration with medium significance for all these feelings. HCWs working in the *ad hoc* created COVID-19 units experienced more often the psychological distress (anxiety 78.7%, tiredness 93.6%, frustration 81.9%), than those working in anesthesia, reanimation and intensive care unit (anxiety 68.7%, tiredness, 78.1% frustration 53.1%). Table 5 summarizes multinomial logistic regressions omnibus Likelihood Ratio tests for psychological impact category encompassing negative feelings and demographic factors (all the models coefficients, standard errors and relative significance are reported in the Supplementary Material).

### Multinomial Logistic Regression: Positive Feelings

*Gender* was found to be a good predictor of quiet, solidarity, satisfaction, and trust but not of hope, and pride. Particularly, quiet and satisfaction were the best feelings predicted by this factor with a high significance, followed by solidarity and trust with a medium to low significance. These feelings were more prevalent among females (quiet 79.9%, solidarity 94.8%, satisfaction 83.8%, trust 86.2%). *Age* was predictive of all positive emotions, showing a high significance, except for satisfaction and pride. The > 55-year-old age group experienced more often these feelings (trust 91.2%, hope 94.5%, solidarity 98.6%, quiet 85%) than the > 34-year-old age group (trust 81.5%, hope 85.8%, solidarity 92.5%, quiet 75.4%). As to the *Professional role*, only satisfaction was found to be predicted with a medium to low significance by this factor; this feeling was felt to a greater extent by doctors (86.5%) than nurses (82.1%). Lastly, one low significant relationship was only found between the *Operational unit* and solidarity: HCWs based in anesthesia, reanimation, intensive care units experienced more often this feeling (99.9%) than other units (94.6%). All the other feelings were far from being affected by this factor. However, *Region* did not likely affect the experience of all the positive emotions. This data suggests that HCWs contacted with our questionnaires across Italy experienced the same feelings.

Table 6 summarizes multinomial logistic regressions omnibus Likelihood Ratio tests for psychological impact category encompassing positive feelings and demographic factors (all the models coefficients, standard errors and relative significance are reported in the Supplementary Material).

## Discussion

Since the beginning of the pandemic, HCWs have been called on the frontline to cope with the current global health emergency. The emergency has imposed on them an overwhelming workload and emotional involvement, thus amplifying those psychosocial risk factors that normally characterize the healthcare sector (21–23, 25, 47). The situation was aggravated by the necessary measures adopted by governments to reduce the spread of the virus such as social distancing and quarantine, which significantly affected their emotional stability and which made impossible for them to benefit from the normal support of family members and friends who are known to represent an asset, a protective factor, especially in difficult times (48–51).

Previous studies have shown that frontline HCWs treating COVID-19 patients experienced higher risk of several symptoms such as anxiety, depression, and insomnia as well as negative feelings including tense, scared, angry, sad, afraid, and impressed (13, 25, 32, 33, 52). Italian health workers, for instance, reported a high level of burnout, psychological symptoms, and emotional exhaustion during COVID-19 pandemic (53). Positive feelings, on the other hand, including conscientiousness and self-sacrifice for patients were also reported by HCWs while they were putting their health and live at risk for patients (43, 44). This finding is particularly interesting as positive emotional states have rarely been investigated in HCWs working in similar circumstances.

With the present study we enrich the extant literature by analyzing the nature and the severity of the psychological complaints reported by the HCWs during the first COVID-19 outbreak in Italy, and by identifying possible predictive factors that led to significant differences in experiencing such psychological distress. Furthermore, we analyzed the possible experience of positive emotions to highlight possible protective factors needed for adaptive coping. We carried out multinomial logistic regressions to investigate the relationship between 21 accurately selected emotional states, negative and positive (loneliness, anxiety, irritability, sadness, tiredness, insecurity, apathy, intolerance, frustration, insomnia, fear, impatience, impotence, anger, resignation, pride, satisfaction, trust, hope, solidarity, quiet), and five possible predictor factors (gender, age, region of Italy, professional role, operational unit).

Regarding the negative feelings, we found that more than half of the HCWs experienced all the emotional states investigated at least sometimes, with the exception of apathy (30.5% of the sample). The most frequently felt emotions were tiredness, irritability, anxiety, and sadness respectively. Factors associated with a higher psychological impact included being a woman, living in northern Italy and young age. These results are in line with the recent literature reporting higher levels of psychological distress in women and young adults (9, 18, 54). Our findings have shown that this holds true for HCWs.

More than half of the HCWs also experienced all positive feelings with the most frequently felt being solidarity, a feeling that has also been reported for the general population in different countries during this health emergency (55). Factors associated with higher experience of these emotional states included female gender and older age.

Differently from other studies, in which the role of health workers and the type of unit mattered (32, 56), we found that the majority of the negative as well as positive emotional states were experienced almost equally by both doctors and nurses, and independently of the operational unit in which they operated. Our finding highlights the importance of investigating both the working role and unit that led to the psychological discomfort, as it has been done in most studies on this subject to date, and the specific emotions as the distinct, contributing factors.

Our results showed also that, overall, female respondents experienced emotional states, be them negative or positive, more often than men. The prevalence of the psychological impact on women may partly reflect gender differences in self-disclosure and in expressing one's feelings: women have been reported before being more likely than men to report their emotional states, especially the negative ones associated with psychological difficulties (57–59). On the other hand, younger health workers suffered psychological distress more frequently than the older ones who, instead, experienced more positive emotions. This pattern of results observed with HCWs extends the observation during this pandemic that, in the general population, younger adults were subjected to stress, depression and anxiety, while older adults were found to score low on ratings about these measures, thus demonstrating more resilience and higher coping strategies (9, 18, 54). Lastly, territorial differences were found only in the negative emotions of the HCWs operating in northern Italy, as this was the most affected region especially around the time of our data collection.

Our results are in line with the research on the psychological impact caused by the present pandemic on the general population (9, 17, 60–62), as well as with that on a specific category of workers like HCWs (25, 26, 32, 53, 63–65). This study has several other merits. First, we considered differences in emotions experienced by respondents depending on their professional role, work units or departments, and regional territory, in addition to the other most studied demographic variables such as gender and age. Second, we investigated a broad spectrum of negative emotional states to better grasp for the complexity of the psychological experience during the pandemic. Third, we also analyzed positive feelings, often overlooked, as they can help us to better characterize to the full the HCWs emotional experience during the pandemic.

This study suffers from a number of weaknesses. First, we administered questionnaires that were not validated and contained one-item scale. This choice was motivated by our purposes to survey a broad spectrum of emotions of HCWs while the health emergency that imposed heavy timing and accessibility limits. Although single-item measures are very useful and accepted in circumstances like ours, with limited time and the need to minimize the burden of respondents who were already highly busy, suffering and tired, the use of multiple items is generally suggested because it helps to average out errors and specificities that are inherent in single items, thus leading to increased reliability and construct validity. Second, being a self-report, this questionnaire may suffer from social desirability bias which can confound relationships among the variables of interest, particularly regarding negative emotions, by obscuring or producing them artificially despite having been guaranteed anonymity. Third, another risk for self-report measures is the recall bias, especially when respondents have experienced heavy emotional events, as in our case, that may have distorted their memories by leading to an over or under-estimation of positive and/or negative past emotional experiences. However, since the questionnaire was spread a few months after the start of the health emergency, with questions relating to the recent and also current experience of the respondents, we believe the influence of this bias is low, even if it should be taken into account. Fourth, we spread the questionnaire in a period in which the workload was overwhelming for the respondents. This factor might have affected the participation, as well as the representativity of the sample which leans toward the female gender. In future studies more representative and balanced samples should be involved. As an exploratory study, the data were analyzed without multiplicity adjustment and the results were interpreted primarily as preliminary insights (66); therefore, future confirmatory studies are needed to test specific and definitive hypotheses. Moreover, the cross-sectional nature of the study and the lack of longitudinal follow-up do not allow inferences about the causal relationships among the variables, and the long-term consequences of the psychological impact we documented.

## Conclusion

Our findings suggest that more than half of HCWs experienced psychological distress during the first COVID-19 outbreak in Italy, and that the factors associated with higher psychological impact included being female, young and living in northern Italy. The most frequently negative emotions reported were tiredness, irritability, anxiety, and sadness. However, positive feelings were also experienced, first of all solidarity, especially by women and older people. Despite some limitations, we believe this study can be very useful as a contribution to the current literature on the psychological effects of this pandemic on health workers. Moreover our findings can inform future policies aimed at providing more tailored and effective psychological interventions in the ongoing and future emergencies. Noteworthy, the HCWs' burdens and mental sufferance affect not only their own health, but pose great concern on their families and friends, as well as on their patients (67). The emergency has been amplifying psychosocial risk factors, already present in the healthcare sector (21–23), that are associated with staff's working conditions, safety and health. Consequently, in addition to support interventions, it would be desirable that hospitals consider adopting work-family policies to foster HCWs' psychological wellbeing by improving their resilience and coping strategies (68). It has become ever so evident that the safeguard of these professionals is necessary and urgent to promote a positive quality of life for them and for the people they come into contact with.

## Data Availability Statement

The datasets presented in this study can be found in online repositories together with the R syntax file used for the analysis, and the questionnaires. The names of the repository/repositories and accession number(s) can be found below: OSF repository, https://osf.io/h8xyu/.

## Ethics Statement

The studies involving human participants were reviewed and approved by Ethics Committee, International School for Advanced Studies (SISSA). The patients/participants provided their written informed consent to participate in this study.

## Author Contributions

EP conducted the data analyses and wrote the first draft of the manuscript. All authors made important contributions to its final version, have read and approved the final version of the manuscript, and contributed to the study design of the study and its implementation.

## Conflict of Interest

The authors declare that the research was conducted in the absence of any commercial or financial relationships that could be construed as a potential conflict of interest.

## Publisher's Note

All claims expressed in this article are solely those of the authors and do not necessarily represent those of their affiliated organizations, or those of the publisher, the editors and the reviewers. Any product that may be evaluated in this article, or claim that may be made by its manufacturer, is not guaranteed or endorsed by the publisher.
