# Supplementary material for: Psychological Impact in Healthcare Workers During Emergencies: The Italian Experience With COVID-19 First Wave
Source: Front Psychiatry. 2022 Mar 21;13:818674. doi: 10.3389/fpsyt.2022.818674 (PMC8977468; doi:10.3389/fpsyt.2022.818674)
Supplement: Supplementary file 1 [file Table_1.DOCX]

Supplementary Material

| **Supplementary table 1.** Models coefficients, standard errors and relative significance for Loneliness | | | | | |
| --- | --- | --- | --- | --- | --- |
| **Loneliness** | | | | | |
| **Predictors** | **Estimate** | **std. Error** | **z-Stat** | ***P*-value** | **Response** |
| (Intercept) | 0.29 | 0.3 | 0.99 | 0.321 | Sometimes |
| Gender [M] | -0.3 | 0.22 | -1.4 | 0.161 | Sometimes |
| Age35-54 | -0.75 | 0.24 | -3.1 | **0.002** | Sometimes |
| Age [>=55] | -1 | 0.29 | -3.51 | **<0.001** | Sometimes |
| Region [North] | 0.53 | 0.22 | 2.43 | **0.015** | Sometimes |
| Role [Nurse] | -0.3 | 0.23 | -1.32 | 0.187 | Sometimes |
| Unit [Anesthesia/Reanimation/Inten.care] | -0.78 | 0.43 | -1.81 | 0.071 | Sometimes |
| Unit [New COVID unit] | 0.36 | 0.26 | 1.37 | 0.172 | Sometimes |
|  |  |  |  |  |  |
| (Intercept) | -0.09 | 0.37 | -0.26 | 0.798 | Often |
| Gender [M] | -0.84 | 0.31 | -2.67 | **0.008** | Often |
| Age35-54 | -1.13 | 0.31 | -3.68 | **<0.001** | Often |
| Age [>=55] | -1.25 | 0.37 | -3.39 | **0.001** | Often |
| Region [North] | 0.6 | 0.3 | 2.02 | **0.043** | Often |
| Role [Nurse] | -0.42 | 0.3 | -1.43 | 0.153 | Often |
| Unit [Anesthesia/Reanimation/Inten.care] | -1.26 | 0.67 | -1.88 | 0.059 | Often |
| Unit [New COVID unit] | -0.02 | 0.36 | -0.05 | 0.963 | Often |
|  |  |  |  |  |  |
| (Intercept) | -2.57 | 1.01 | -2.55 | **0.011** | Almost always or always |
| Gender [M] | -0.48 | 0.83 | -0.57 | 0.568 | Almost always or always |
| Age35-54 | -1.41 | 0.7 | -2.01 | **0.044** | Almost always or always |
| Age [>=55] | -14.21 | 423.78 | -0.03 | 0.973 | Almost always or always |
| Region [North] | 0.77 | 0.84 | 0.92 | 0.357 | Almost always or always |
| Role [Nurse] | 0.07 | 0.86 | 0.08 | 0.934 | Almost always or always |
| Unit [Anesthesia/Reanimation/Inten.care] | -113.09 | NaN | NaN | NaN | Almost always or always |
| Unit [New COVID unit] | 0.3 | 0.85 | 0.35 | 0.727 | Almost always or always |

*Significant P values are highlighted in bold.*

| **Supplementary table 2.** Models coefficients, standard errors and relative significance for Anxiety | | | | | |
| --- | --- | --- | --- | --- | --- |
| **Anxiety** | | | | | |
| **Predictors** | **Estimate** | **std. Error** | **z-Stat** | ***P*-value** | **Response** |
| (Intercept) | 1.56 | 0.36 | 4.35 | **<0.001** | Sometimes |
| Gender [M] | -1 | 0.24 | -4.16 | **<0.001** | Sometimes |
| Age35-54 | -0.62 | 0.29 | -2.13 | **0.033** | Sometimes |
| Age [>=55] | -0.6 | 0.33 | -1.81 | 0.071 | Sometimes |
| Region [North] | 0.51 | 0.24 | 2.11 | **0.035** | Sometimes |
| Role [Nurse] | -0.4 | 0.26 | -1.52 | 0.129 | Sometimes |
| Unit [Anesthesia/Reanimation/Inten.care] | -0.54 | 0.45 | -1.2 | 0.229 | Sometimes |
| Unit [New COVID unit] | -0.38 | 0.32 | -1.18 | 0.236 | Sometimes |
|  |  |  |  |  |  |
| (Intercept) | 0.75 | 0.41 | 1.85 | 0.064 | Often |
| Gender [M] | -1.05 | 0.29 | -3.68 | **<0.001** | Often |
| Age35-54 | -0.68 | 0.32 | -2.12 | **0.034** | Often |
| Age [>=55] | -1.24 | 0.39 | -3.13 | **0.002** | Often |
| Region [North] | 0.85 | 0.3 | 2.89 | **0.004** | Often |
| Role [Nurse] | -0.52 | 0.3 | -1.72 | 0.085 | Often |
| Unit [Anesthesia/Reanimation/Inten.care] | -1.03 | 0.6 | -1.72 | 0.086 | Often |
| Unit [New COVID unit] | 0.33 | 0.34 | 0.99 | 0.324 | Often |
|  |  |  |  |  |  |
| (Intercept) | -0.14 | 0.59 | -0.24 | 0.807 | Almost always or always |
| Gender [M] | -1.84 | 0.6 | -3.08 | **0.002** | Almost always or always |
| Age35-54 | -1.27 | 0.52 | -2.43 | **0.015** | Almost always or always |
| Age [>=55] | -0.94 | 0.6 | -1.55 | 0.12 | Almost always or always |
| Region [North] | -0.18 | 0.48 | -0.38 | 0.701 | Almost always or always |
| Role [Nurse] | -0.33 | 0.5 | -0.66 | 0.509 | Almost always or always |
| Unit [Anesthesia/Reanimation/Inten.care] | -0.56 | 1.12 | -0.5 | 0.615 | Almost always or always |
| Unit [New COVID unit] | 1.03 | 0.51 | 2.01 | **0.045** | Almost always or always |

*Significant P values are highlighted in bold.*

| **Supplementary table 3.** Models coefficients, standard errors and relative significance for Irritability | | | | | |
| --- | --- | --- | --- | --- | --- |
| **Irritability** | | | | | |
| **Predictors** | **Estimate** | **std. Error** | **z-Stat** | ***P*-value** | **Response** |
| (Intercept) | 1.22 | 0.39 | 3.09 | **0.002** | Sometimes |
| Gender [M] | -0.43 | 0.26 | -1.65 | 0.099 | Sometimes |
| Age35-54 | -0.34 | 0.32 | -1.07 | 0.287 | Sometimes |
| Age [>=55] | -0.43 | 0.36 | -1.2 | 0.231 | Sometimes |
| Region [North] | 0.78 | 0.27 | 2.92 | **0.003** | Sometimes |
| Role [Nurse] | -0.53 | 0.29 | -1.84 | 0.066 | Sometimes |
| Unit [Anesthesia/Reanimation/Inten.care] | -1.07 | 0.48 | -2.22 | **0.026** | Sometimes |
| Unit [New COVID unit] | 0.1 | 0.37 | 0.28 | 0.78 | Sometimes |
|  |  |  |  |  |  |
| (Intercept) | 1.55 | 0.4 | 3.86 | **<0.001** | Often |
| Gender [M] | -0.9 | 0.29 | -3.16 | **0.002** | Often |
| Age35-54 | -0.61 | 0.32 | -1.87 | 0.061 | Often |
| Age [>=55] | -0.87 | 0.38 | -2.28 | **0.023** | Often |
| Region [North] | 0.3 | 0.28 | 1.06 | 0.288 | Often |
| Role [Nurse] | -0.55 | 0.3 | -1.8 | 0.072 | Often |
| Unit [Anesthesia/Reanimation/Inten.care] | -0.89 | 0.52 | -1.73 | 0.084 | Often |
| Unit [New COVID unit] | 0.45 | 0.37 | 1.2 | 0.23 | Often |
|  |  |  |  |  |  |
| (Intercept) | -0.32 | 0.65 | -0.49 | 0.625 | Almost always or always |
| Gender [M] | -0.3 | 0.5 | -0.59 | 0.556 | Almost always or always |
| Age35-54 | -1.08 | 0.5 | -2.14 | **0.032** | Almost always or always |
| Age [>=55] | -3.04 | 1.1 | -2.77 | **0.006** | Almost always or always |
| Region [North] | 0.58 | 0.52 | 1.11 | 0.267 | Almost always or always |
| Role [Nurse] | -0.52 | 0.53 | -0.98 | 0.325 | Almost always or always |
| Unit [Anesthesia/Reanimation/Inten.care] | -1.58 | 1.13 | -1.4 | 0.16 | Almost always or always |
| Unit [New COVID unit] | 0.29 | 0.61 | 0.47 | 0.637 | Almost always or always |

*Significant P values are highlighted in bold.*

| **Supplementary table 4.** Models coefficients, standard errors and relative significance for Sadness | | | | | |
| --- | --- | --- | --- | --- | --- |
| **Sadness** | | | | | |
| **Predictors** | **Estimate** | **std. Error** | **z-Stat** | ***P*-value** | **Response** |
| (Intercept) | 1.09 | 0.34 | 3.24 | **0.001** | Sometimes |
| Gender [M] | -0.55 | 0.23 | -2.35 | **0.019** | Sometimes |
| Age35-54 | -0.43 | 0.28 | -1.57 | 0.117 | Sometimes |
| Age [>=55] | -0.76 | 0.31 | -2.41 | **0.016** | Sometimes |
| Region [North] | 0.34 | 0.24 | 1.44 | 0.151 | Sometimes |
| Role [Nurse] | -0.24 | 0.25 | -0.97 | 0.332 | Sometimes |
| Unit [Anesthesia/Reanimation/Inten.care] | -0.11 | 0.45 | -0.24 | 0.808 | Sometimes |
| Unit [New COVID unit] | 0.42 | 0.32 | 1.31 | 0.191 | Sometimes |
|  |  |  |  |  |  |
| (Intercept) | 0.48 | 0.38 | 1.27 | 0.205 | Often |
| Gender [M] | -1.01 | 0.28 | -3.55 | **<0.001** | Often |
| Age35-54 | -0.39 | 0.31 | -1.25 | 0.21 | Often |
| Age [>=55] | -0.69 | 0.36 | -1.91 | 0.056 | Often |
| Region [North] | 0.42 | 0.28 | 1.52 | 0.129 | Often |
| Role [Nurse] | -0.24 | 0.29 | -0.83 | 0.404 | Often |
| Unit [Anesthesia/Reanimation/Inten.care] | -0.43 | 0.57 | -0.75 | 0.45 | Often |
| Unit [New COVID unit] | 0.64 | 0.35 | 1.81 | 0.07 | Often |
|  |  |  |  |  |  |
| (Intercept) | -0.88 | 0.66 | -1.33 | 0.185 | Almost always or always |
| Gender [M] | -1.14 | 0.61 | -1.88 | 0.06 | Almost always or always |
| Age35-54 | -0.54 | 0.54 | -1 | 0.318 | Almost always or always |
| Age [>=55] | -1.41 | 0.75 | -1.87 | 0.061 | Almost always or always |
| Region [North] | 0.54 | 0.54 | 1 | 0.315 | Almost always or always |
| Role [Nurse] | -0.45 | 0.55 | -0.82 | 0.411 | Almost always or always |
| Unit [Anesthesia/Reanimation/Inten.care] | -12.9 | 0 | -9577100 | **<0.001** | Almost always or always |
| Unit [New COVID unit] | -0.49 | 0.8 | -0.6 | 0.546 | Almost always or always |

*Significant P values are highlighted in bold.*

| **Supplementary table 5.** Models coefficients, standard errors and relative significance for Tiredness | | | | | |
| --- | --- | --- | --- | --- | --- |
| **Tiredness** | | | | | |
| **Predictors** | **Estimate** | **std. Error** | **z-Stat** | ***P*-value** | **Response** |
| (Intercept) | 1.06 | 0.43 | 2.46 | **0.014** | Sometimes |
| Gender [M] | -0.52 | 0.3 | -1.73 | 0.083 | Sometimes |
| Age35-54 | 0.45 | 0.36 | 1.27 | 0.206 | Sometimes |
| Age [>=55] | -0.11 | 0.39 | -0.28 | 0.782 | Sometimes |
| Region [North] | 0.56 | 0.3 | 1.84 | 0.065 | Sometimes |
| Role [Nurse] | -0.32 | 0.34 | -0.95 | 0.342 | Sometimes |
| Unit [Anesthesia/Reanimation/Inten.care] | -0.57 | 0.53 | -1.07 | 0.283 | Sometimes |
| Unit [New COVID unit] | 0.61 | 0.51 | 1.19 | 0.236 | Sometimes |
|  |  |  |  |  |  |
| (Intercept) | 1.38 | 0.43 | 3.21 | **0.001** | Often |
| Gender [M] | -0.99 | 0.31 | -3.21 | **0.001** | Often |
| Age35-54 | 0.02 | 0.36 | 0.05 | 0.961 | Often |
| Age [>=55] | -0.6 | 0.4 | -1.52 | 0.127 | Often |
| Region [North] | 0.75 | 0.31 | 2.42 | **0.016** | Often |
| Role [Nurse] | -0.34 | 0.34 | -0.99 | 0.322 | Often |
| Unit [Anesthesia/Reanimation/Inten.care] | -0.71 | 0.54 | -1.32 | 0.188 | Often |
| Unit [New COVID unit] | 1.01 | 0.51 | 1.99 | **0.047** | Often |
|  |  |  |  |  |  |
| (Intercept) | -0.11 | 0.64 | -0.17 | 0.863 | Almost always or always |
| Gender [M] | -1.63 | 0.52 | -3.12 | **0.002** | Almost always or always |
| Age35-54 | 0.07 | 0.51 | 0.13 | 0.893 | Almost always or always |
| Age [>=55] | -1.15 | 0.64 | -1.81 | 0.07 | Almost always or always |
| Region [North] | 1.87 | 0.54 | 3.45 | **0.001** | Almost always or always |
| Role [Nurse] | -1.79 | 0.49 | -3.68 | **<0.001** | Almost always or always |
| Unit [Anesthesia/Reanimation/Inten.care] | -14.01 | 0 | -6667287 | **<0.001** | Almost always or always |
| Unit [New COVID unit] | 0.47 | 0.66 | 0.71 | 0.475 | Almost always or always |

*Significant P values are highlighted in bold.*

| **Supplementary table 6.** Models coefficients, standard errors and relative significance for Insecurity | | | | | |
| --- | --- | --- | --- | --- | --- |
| **Insecurity** | | | | | |
| **Predictors** | **Estimate** | **std. Error** | **z-Stat** | ***P*-value** | **Response** |
| (Intercept) | 0.91 | 0.33 | 2.78 | **0.005** | Sometimes |
| Gender [M] | -0.75 | 0.23 | -3.3 | **0.001** | Sometimes |
| Age35-54 | -0.58 | 0.27 | -2.12 | **0.034** | Sometimes |
| Age [>=55] | -0.51 | 0.31 | -1.67 | 0.095 | Sometimes |
| Region [North] | 0.66 | 0.23 | 2.88 | **0.004** | Sometimes |
| Role [Nurse] | -0.11 | 0.25 | -0.44 | 0.657 | Sometimes |
| Unit [Anesthesia/Reanimation/Inten.care] | -0.42 | 0.44 | -0.97 | 0.332 | Sometimes |
| Unit [New COVID unit] | 0.14 | 0.31 | 0.47 | 0.636 | Sometimes |
|  |  |  |  |  |  |
| (Intercept) | 0.4 | 0.38 | 1.04 | 0.3 | Often |
| Gender [M] | -1.16 | 0.3 | -3.89 | **<0.001** | Often |
| Age35-54 | -0.61 | 0.31 | -1.94 | 0.052 | Often |
| Age [>=55] | -1.3 | 0.4 | -3.28 | **0.001** | Often |
| Region [North] | 0.87 | 0.29 | 2.97 | **0.003** | Often |
| Role [Nurse] | -0.48 | 0.3 | -1.64 | 0.102 | Often |
| Unit [Anesthesia/Reanimation/Inten.care] | -0.8 | 0.59 | -1.35 | 0.178 | Often |
| Unit [New COVID unit] | 0.46 | 0.35 | 1.33 | 0.183 | Often |
|  |  |  |  |  |  |
| (Intercept) | -1.99 | 0.87 | -2.28 | **0.023** | Almost always or always |
| Gender [M] | -0.47 | 0.64 | -0.74 | 0.462 | Almost always or always |
| Age35-54 | -0.82 | 0.59 | -1.4 | 0.163 | Almost always or always |
| Age [>=55] | -14.87 | 0 | -10730284 | **<0.001** | Almost always or always |
| Region [North] | 1.04 | 0.69 | 1.5 | 0.135 | Almost always or always |
| Role [Nurse] | 0.14 | 0.71 | 0.2 | 0.841 | Almost always or always |
| Unit [Anesthesia/Reanimation/Inten.care] | -13.62 | 0 | -19938607 | **<0.001** | Almost always or always |
| Unit [New COVID unit] | -0.22 | 0.82 | -0.27 | 0.789 | Almost always or always |

*Significant P values are highlighted in bold.*

| **Supplementary table 7.** Models coefficients, standard errors and relative significance for Intolerance | | | | | |
| --- | --- | --- | --- | --- | --- |
| **Intolerance** | | | | | |
| **Predictors** | **Estimate** | **std. Error** | **z-Stat** | ***P*-value** | **Response** |
| (Intercept) | 0.22 | 0.3 | 0.74 | 0.457 | Sometimes |
| Gender [M] | -0.47 | 0.22 | -2.12 | **0.034** | Sometimes |
| Age35-54 | -0.13 | 0.25 | -0.53 | 0.593 | Sometimes |
| Age [>=55] | -0.45 | 0.29 | -1.58 | 0.114 | Sometimes |
| Region [North] | 0.29 | 0.22 | 1.33 | 0.185 | Sometimes |
| Role [Nurse] | 0.09 | 0.23 | 0.4 | 0.689 | Sometimes |
| Unit [Anesthesia/Reanimation/Inten.care] | -0.17 | 0.44 | -0.38 | 0.7 | Sometimes |
| Unit [New COVID unit] | -0.05 | 0.27 | -0.18 | 0.857 | Sometimes |
|  |  |  |  |  |  |
| (Intercept) | -0.37 | 0.36 | -1.03 | 0.303 | Often |
| Gender [M] | -0.08 | 0.26 | -0.32 | 0.751 | Often |
| Age35-54 | -0.33 | 0.29 | -1.12 | 0.261 | Often |
| Age [>=55] | -0.89 | 0.35 | -2.56 | **0.011** | Often |
| Region [North] | 0.84 | 0.28 | 2.96 | **0.003** | Often |
| Role [Nurse] | -0.54 | 0.27 | -1.99 | **0.046** | Often |
| Unit [Anesthesia/Reanimation/Inten.care] | -0.58 | 0.54 | -1.06 | 0.287 | Often |
| Unit [New COVID unit] | -0.17 | 0.32 | -0.52 | 0.601 | Often |
|  |  |  |  |  |  |
| (Intercept) | -2.01 | 0.8 | -2.53 | **0.011** | Almost always or always |
| Gender [M] | -2.16 | 1.07 | -2.01 | **0.044** | Almost always or always |
| Age35-54 | -0.99 | 0.58 | -1.7 | 0.089 | Almost always or always |
| Age [>=55] | -2.06 | 1.1 | -1.88 | 0.06 | Almost always or always |
| Region [North] | 0.4 | 0.63 | 0.63 | 0.53 | Almost always or always |
| Role [Nurse] | 0.44 | 0.7 | 0.62 | 0.534 | Almost always or always |
| Unit [Anesthesia/Reanimation/Inten.care] | 0.86 | 0.89 | 0.97 | 0.332 | Almost always or always |
| Unit [New COVID unit] | -0.27 | 0.82 | -0.33 | 0.743 | Almost always or always |

*Significant P values are highlighted in bold.*

| **Supplementary table 8.** Models coefficients, standard errors and relative significance for Frustration | | | | | |
| --- | --- | --- | --- | --- | --- |
| **Frustration** | | | | | |
| **Predictors** | **Estimate** | **std. Error** | **z-Stat** | ***P*-value** | **Response** |
| (Intercept) | -0.04 | 0.31 | -0.11 | 0.91 | Sometimes |
| Gender [M] | -0.1 | 0.23 | -0.44 | 0.66 | Sometimes |
| Age35-54 | -0.03 | 0.26 | -0.12 | 0.902 | Sometimes |
| Age [>=55] | -0.02 | 0.3 | -0.07 | 0.945 | Sometimes |
| Region [North] | 0.62 | 0.23 | 2.73 | **0.006** | Sometimes |
| Role [Nurse] | -0.35 | 0.24 | -1.44 | 0.151 | Sometimes |
| Unit [Anesthesia/Reanimation/Inten.care] | -0.51 | 0.43 | -1.19 | 0.233 | Sometimes |
| Unit [New COVID unit] | 0.69 | 0.32 | 2.18 | **0.029** | Sometimes |
|  |  |  |  |  |  |
| (Intercept) | -0.16 | 0.34 | -0.46 | 0.643 | Often |
| Gender [M] | -0.44 | 0.27 | -1.65 | 0.1 | Often |
| Age35-54 | -0.44 | 0.28 | -1.57 | 0.117 | Often |
| Age [>=55] | -0.67 | 0.34 | -1.94 | 0.052 | Often |
| Region [North] | 0.83 | 0.27 | 3.08 | **0.002** | Often |
| Role [Nurse] | -0.43 | 0.27 | -1.57 | 0.117 | Often |
| Unit [Anesthesia/Reanimation/Inten.care] | -1.05 | 0.56 | -1.89 | 0.058 | Often |
| Unit [New COVID unit] | 0.81 | 0.34 | 2.39 | **0.017** | Often |
|  |  |  |  |  |  |
| (Intercept) | -2.11 | 0.73 | -2.89 | **0.004** | Almost always or always |
| Gender [M] | -2.25 | 1.05 | -2.14 | **0.032** | Almost always or always |
| Age35-54 | -0.86 | 0.52 | -1.66 | 0.098 | Almost always or always |
| Age [>=55] | -1.03 | 0.72 | -1.44 | 0.151 | Almost always or always |
| Region [North] | 1.04 | 0.6 | 1.72 | 0.086 | Almost always or always |
| Role [Nurse] | 0.2 | 0.62 | 0.32 | 0.746 | Almost always or always |
| Unit [Anesthesia/Reanimation/Inten.care] | -12.6 | 404.57 | -0.03 | 0.975 | Almost always or always |
| Unit [New COVID unit] | 0.89 | 0.59 | 1.5 | 0.133 | Almost always or always |

*Significant P values are highlighted in bold.*

| **Supplementary table 9.** Models coefficients, standard errors and relative significance for Insomnia | | | | | |
| --- | --- | --- | --- | --- | --- |
| **Insomnia** | | | | | |
| **Predictors** | **Estimate** | **std. Error** | **z-Stat** | ***P*-value** | **Response** |
| (Intercept) | -0.13 | 0.3 | -0.43 | 0.669 | Sometimes |
| Gender [M] | -0.77 | 0.23 | -3.32 | **0.001** | Sometimes |
| Age35-54 | 0 | 0.25 | 0.01 | 0.991 | Sometimes |
| Age [>=55] | -0.06 | 0.3 | -0.21 | 0.837 | Sometimes |
| Region [North] | 0.21 | 0.23 | 0.94 | 0.349 | Sometimes |
| Role [Nurse] | -0.22 | 0.24 | -0.92 | 0.356 | Sometimes |
| Unit [Anesthesia/Reanimation/Inten.care] | -0.59 | 0.51 | -1.16 | 0.247 | Sometimes |
| Unit [New COVID unit] | 0.42 | 0.3 | 1.42 | 0.156 | Sometimes |
|  |  |  |  |  |  |
| (Intercept) | -0.85 | 0.36 | -2.34 | **0.019** | Often |
| Gender [M] | -1.14 | 0.29 | -3.95 | **<0.001** | Often |
| Age35-54 | 0.14 | 0.29 | 0.47 | 0.641 | Often |
| Age [>=55] | -0.02 | 0.35 | -0.06 | 0.949 | Often |
| Region [North] | 0.52 | 0.28 | 1.84 | 0.066 | Often |
| Role [Nurse] | -0.27 | 0.28 | -0.99 | 0.323 | Often |
| Unit [Anesthesia/Reanimation/Inten.care] | -0.02 | 0.52 | -0.03 | 0.976 | Often |
| Unit [New COVID unit] | 0.84 | 0.32 | 2.63 | **0.008** | Often |
|  |  |  |  |  |  |
| (Intercept) | -2.04 | 0.55 | -3.67 | **<0.001** | Almost always or always |
| Gender [M] | -1.1 | 0.44 | -2.5 | **0.013** | Almost always or always |
| Age35-54 | -0.47 | 0.42 | -1.13 | 0.26 | Almost always or always |
| Age [>=55] | -0.18 | 0.49 | -0.38 | 0.705 | Almost always or always |
| Region [North] | 0.82 | 0.46 | 1.78 | 0.076 | Almost always or always |
| Role [Nurse] | 0.01 | 0.42 | 0.02 | 0.988 | Almost always or always |
| Unit [Anesthesia/Reanimation/Inten.care] | 0.24 | 0.7 | 0.35 | 0.726 | Almost always or always |
| Unit [New COVID unit] | 0.96 | 0.44 | 2.18 | **0.030** | Almost always or always |

*Significant P values are highlighted in bold.*

| **Supplementary table 10.** Models coefficients, standard errors and relative significance for Apathy | | | | | |
| --- | --- | --- | --- | --- | --- |
| **Apathy** | | | | | |
| **Predictors** | **Estimate** | **std. Error** | **z-Stat** | ***P*-value** | **Response** |
| (Intercept) | -0.97 | 0.31 | -3.11 | **0.002** | Sometimes |
| Gender [M] | 0.07 | 0.24 | 0.29 | 0.769 | Sometimes |
| Age35-54 | -0.29 | 0.26 | -1.12 | 0.263 | Sometimes |
| Age [>=55] | -0.23 | 0.31 | -0.75 | 0.455 | Sometimes |
| Region [North] | -0.23 | 0.24 | -0.95 | 0.34 | Sometimes |
| Role [Nurse] | 0.08 | 0.25 | 0.34 | 0.734 | Sometimes |
| Unit [Anesthesia/Reanimation/Inten.care] | 0.05 | 0.47 | 0.11 | 0.912 | Sometimes |
| Unit [New COVID unit] | 0.23 | 0.28 | 0.82 | 0.414 | Sometimes |
|  |  |  |  |  |  |
| (Intercept) | -1.48 | 0.46 | -3.22 | **0.001** | Often |
| Gender [M] | 0.03 | 0.38 | 0.07 | 0.942 | Often |
| Age35-54 | -1.13 | 0.4 | -2.81 | **0.005** | Often |
| Age [>=55] | -0.96 | 0.46 | -2.1 | **0.036** | Often |
| Region [North] | 0.52 | 0.4 | 1.28 | 0.2 | Often |
| Role [Nurse] | -0.75 | 0.38 | -1.98 | **0.048** | Often |
| Unit [Anesthesia/Reanimation/Inten.care] | -0.35 | 0.68 | -0.51 | 0.608 | Often |
| Unit [New COVID unit] | -0.29 | 0.49 | -0.59 | 0.557 | Often |
|  |  |  |  |  |  |
| (Intercept) | -4.41 | 1.1 | -4.01 | **<0.001** | Almost always or always |
| Gender [M] | 1.37 | 0.64 | 2.13 | **0.033** | Almost always or always |
| Age35-54 | -1.69 | 0.71 | -2.38 | **0.017** | Almost always or always |
| Age [>=55] | -1.9 | 1.12 | -1.7 | 0.089 | Almost always or always |
| Region [North] | 0.67 | 0.82 | 0.82 | 0.414 | Almost always or always |
| Role [Nurse] | 1.03 | 0.85 | 1.21 | 0.226 | Almost always or always |
| Unit [Anesthesia/Reanimation/Inten.care] | -12.82 | 469.99 | -0.03 | 0.978 | Almost always or always |
| Unit [New COVID unit] | 0.52 | 0.72 | 0.72 | 0.47 | Almost always or always |

*Significant P values are highlighted in bold.*

| **Supplementary table 11.** Models coefficients, standard errors and relative significance for Fear | | | | | |
| --- | --- | --- | --- | --- | --- |
| **Fear** | | | | | |
| **Predictors** | **Estimate** | **std. Error** | **z-Stat** | ***P*-value** | **Response** |
| (Intercept) | 0.61 | 0.29 | 2.08 | **0.037** | Sometimes |
| Gender [M] | -0.61 | 0.21 | -2.9 | **0.004** | Sometimes |
| Age35-54 | -0.34 | 0.24 | -1.44 | 0.149 | Sometimes |
| Age [>=55] | -0.48 | 0.28 | -1.73 | 0.084 | Sometimes |
| Region [North] | 0.09 | 0.22 | 0.44 | 0.663 | Sometimes |
| Role [Nurse] | -0.01 | 0.22 | -0.03 | 0.972 | Sometimes |
| Unit [Anesthesia/Reanimation/Inten.care] | -0.49 | 0.4 | -1.21 | 0.225 | Sometimes |
| Unit [New COVID unit] | 0.35 | 0.27 | 1.3 | 0.194 | Sometimes |
|  |  |  |  |  |  |
| (Intercept) | -0.46 | 0.4 | -1.15 | 0.249 | Often |
| Gender [M] | -1.44 | 0.36 | -4.02 | **<0.001** | Often |
| Age35-54 | 0.11 | 0.34 | 0.33 | 0.745 | Often |
| Age [>=55] | -0.22 | 0.41 | -0.53 | 0.596 | Often |
| Region [North] | -0.2 | 0.3 | -0.67 | 0.504 | Often |
| Role [Nurse] | 0.03 | 0.32 | 0.09 | 0.928 | Often |
| Unit [Anesthesia/Reanimation/Inten.care] | -0.96 | 0.78 | -1.22 | 0.223 | Often |
| Unit [New COVID unit] | 0.45 | 0.36 | 1.24 | 0.215 | Often |
|  |  |  |  |  |  |
| (Intercept) | -1.84 | 0.81 | -2.28 | **0.023** | Almost always or always |
| Gender [M] | -1.25 | 0.8 | -1.55 | 0.12 | Almost always or always |
| Age35-54 | -0.61 | 0.64 | -0.95 | 0.343 | Almost always or always |
| Age [>=55] | -0.96 | 0.89 | -1.09 | 0.277 | Almost always or always |
| Region [North] | -0.33 | 0.62 | -0.53 | 0.596 | Almost always or always |
| Role [Nurse] | 0.44 | 0.73 | 0.6 | 0.548 | Almost always or always |
| Unit [Anesthesia/Reanimation/Inten.care] | -12.33 | 0 | -6393140 | **<0.001** | Almost always or always |
| Unit [New COVID unit] | -0.67 | 1.08 | -0.62 | 0.535 | Almost always or always |

*Significant P values are highlighted in bold.*

| **Supplementary table 12.** Models coefficients, standard errors and relative significance for Impatience | | | | | |
| --- | --- | --- | --- | --- | --- |
| **Impatience** | | | | | |
| **Predictors** | **Estimate** | **std. Error** | **z-Stat** | ***P*-value** | **Response** |
| (Intercept) | 0.05 | 0.29 | 0.18 | 0.859 | Sometimes |
| Gender [M] | 0.07 | 0.21 | 0.3 | 0.761 | Sometimes |
| Age35-54 | -0.2 | 0.23 | -0.84 | 0.401 | Sometimes |
| Age [>=55] | 0.07 | 0.27 | 0.25 | 0.805 | Sometimes |
| Region [North] | 0.43 | 0.21 | 2 | **0.045** | Sometimes |
| Role [Nurse] | -0.36 | 0.22 | -1.58 | 0.113 | Sometimes |
| Unit [Anesthesia/Reanimation/Inten.care] | -0.65 | 0.44 | -1.48 | 0.138 | Sometimes |
| Unit [New COVID unit] | 0.01 | 0.26 | 0.02 | 0.983 | Sometimes |
|  |  |  |  |  |  |
| (Intercept) | -0.5 | 0.37 | -1.34 | 0.18 | Often |
| Gender [M] | 0.18 | 0.29 | 0.62 | 0.536 | Often |
| Age35-54 | -0.34 | 0.31 | -1.12 | 0.264 | Often |
| Age [>=55] | -0.89 | 0.4 | -2.23 | **0.026** | Often |
| Region [North] | 0.54 | 0.3 | 1.8 | 0.072 | Often |
| Role [Nurse] | -0.96 | 0.3 | -3.22 | **0.001** | Often |
| Unit [Anesthesia/Reanimation/Inten.care] | -0.36 | 0.54 | -0.66 | 0.507 | Often |
| Unit [New COVID unit] | -0.07 | 0.35 | -0.2 | 0.843 | Often |
|  |  |  |  |  |  |
| (Intercept) | -2.59 | 0.87 | -2.97 | **0.003** | Almost always or always |
| Gender [M] | -0.31 | 0.7 | -0.44 | 0.662 | Almost always or always |
| Age35-54 | -0.91 | 0.64 | -1.43 | 0.153 | Almost always or always |
| Age [>=55] | -0.96 | 0.87 | -1.1 | 0.271 | Almost always or always |
| Region [North] | 0.66 | 0.7 | 0.95 | 0.345 | Almost always or always |
| Role [Nurse] | 0.12 | 0.73 | 0.17 | 0.868 | Almost always or always |
| Unit [Anesthesia/Reanimation/Inten.care] | -0.31 | 1.13 | -0.27 | 0.785 | Almost always or always |
| Unit [New COVID unit] | -0.96 | 1.07 | -0.9 | 0.37 | Almost always or always |

*Significant P values are highlighted in bold.*

| **Supplementary table 13.** Models coefficients, standard errors and relative significance for Impotence | | | | | |
| --- | --- | --- | --- | --- | --- |
| **Impotence** | | | | | |
| **Predictors** | **Estimate** | **std. Error** | **z-Stat** | ***P*-value** | **Response** |
| (Intercept) | 0.61 | 0.33 | 1.86 | 0.063 | Sometimes |
| Gender [M] | -0.2 | 0.23 | -0.9 | 0.368 | Sometimes |
| Age35-54 | -0.46 | 0.27 | -1.69 | 0.091 | Sometimes |
| Age [>=55] | -0.44 | 0.31 | -1.42 | 0.155 | Sometimes |
| Region [North] | 0.64 | 0.23 | 2.73 | **0.006** | Sometimes |
| Role [Nurse] | -0.21 | 0.25 | -0.84 | 0.401 | Sometimes |
| Unit [Anesthesia/Reanimation/Inten.care] | -0.46 | 0.44 | -1.04 | 0.297 | Sometimes |
| Unit [New COVID unit] | -0.17 | 0.3 | -0.58 | 0.56 | Sometimes |
|  |  |  |  |  |  |
| (Intercept) | 0.52 | 0.36 | 1.46 | 0.146 | Often |
| Gender [M] | -0.93 | 0.29 | -3.22 | **0.001** | Often |
| Age35-54 | -0.64 | 0.3 | -2.16 | **0.031** | Often |
| Age [>=55] | -1.14 | 0.37 | -3.05 | **0.002** | Often |
| Region [North] | 0.5 | 0.27 | 1.82 | 0.068 | Often |
| Role [Nurse] | -0.29 | 0.28 | -1.02 | 0.307 | Often |
| Unit [Anesthesia/Reanimation/Inten.care] | -0.84 | 0.58 | -1.45 | 0.148 | Often |
| Unit [New COVID unit] | 0.23 | 0.33 | 0.7 | 0.481 | Often |
|  |  |  |  |  |  |
| (Intercept) | -1.65 | 0.65 | -2.55 | **0.011** | Almost always or always |
| Gender [M] | -0.88 | 0.51 | -1.73 | 0.084 | Almost always or always |
| Age35-54 | -0.58 | 0.49 | -1.18 | 0.236 | Almost always or always |
| Age [>=55] | -0.52 | 0.58 | -0.91 | 0.365 | Almost always or always |
| Region [North] | 0.99 | 0.51 | 1.95 | 0.051 | Almost always or always |
| Role [Nurse] | 0.1 | 0.49 | 0.21 | 0.835 | Almost always or always |
| Unit [Anesthesia/Reanimation/Inten.care] | -1.03 | 1.1 | -0.94 | 0.347 | Almost always or always |
| Unit [New COVID unit] | -0.1 | 0.55 | -0.19 | 0.85 | Almost always or always |

*Significant P values are highlighted in bold.*

| **Supplementary table 14.** Models coefficients, standard errors and relative significance for Anger | | | | | |
| --- | --- | --- | --- | --- | --- |
| **Anger** | | | | | |
| **Predictors** | **Estimate** | **std. Error** | **z-Stat** | ***P*-value** | **Response** |
| (Intercept) | 0.44 | 0.31 | 1.41 | 0.159 | Sometimes |
| Gender [M] | -0.34 | 0.22 | -1.53 | 0.126 | Sometimes |
| Age35-54 | -0.23 | 0.25 | -0.91 | 0.361 | Sometimes |
| Age [>=55] | -0.38 | 0.29 | -1.3 | 0.193 | Sometimes |
| Region [North] | 0.56 | 0.23 | 2.47 | **0.014** | Sometimes |
| Role [Nurse] | -0.34 | 0.24 | -1.44 | 0.15 | Sometimes |
| Unit [Anesthesia/Reanimation/Inten.care] | -0.32 | 0.43 | -0.74 | 0.457 | Sometimes |
| Unit [New COVID unit] | -0.14 | 0.28 | -0.51 | 0.611 | Sometimes |
|  |  |  |  |  |  |
| (Intercept) | 0.16 | 0.35 | 0.45 | 0.65 | Often |
| Gender [M] | -0.47 | 0.27 | -1.74 | 0.081 | Often |
| Age35-54 | -0.3 | 0.28 | -1.08 | 0.279 | Often |
| Age [>=55] | -0.92 | 0.36 | -2.6 | **0.009** | Often |
| Region [North] | 0.26 | 0.26 | 0.98 | 0.326 | Often |
| Role [Nurse] | -0.26 | 0.27 | -0.95 | 0.343 | Often |
| Unit [Anesthesia/Reanimation/Inten.care] | -0.51 | 0.54 | -0.94 | 0.348 | Often |
| Unit [New COVID unit] | 0.11 | 0.31 | 0.34 | 0.733 | Often |
|  |  |  |  |  |  |
| (Intercept) | -2.22 | 0.79 | -2.81 | **0.005** | Almost always or always |
| Gender [M] | -0.54 | 0.61 | -0.88 | 0.377 | Almost always or always |
| Age35-54 | -0.45 | 0.57 | -0.8 | 0.424 | Almost always or always |
| Age [>=55] | -0.92 | 0.76 | -1.22 | 0.223 | Almost always or always |
| Region [North] | 0.77 | 0.61 | 1.26 | 0.207 | Almost always or always |
| Role [Nurse] | 0.22 | 0.64 | 0.34 | 0.732 | Almost always or always |
| Unit [Anesthesia/Reanimation/Inten.care] | -12.53 | 397.51 | -0.03 | 0.975 | Almost always or always |
| Unit [New COVID unit] | -0.66 | 0.79 | -0.83 | 0.407 | Almost always or always |

*Significant P values are highlighted in bold.*

| **Supplementary table 15.** Models coefficients, standard errors and relative significance for Resignation | | | | | |
| --- | --- | --- | --- | --- | --- |
| **Resignation** | | | | | |
| **Predictors** | **Estimate** | **std. Error** | **z-Stat** | ***P*-value** | **Response** |
| (Intercept) | 0.14 | 0.29 | 0.48 | 0.628 | Sometimes |
| Gender [M] | -0.19 | 0.22 | -0.89 | 0.372 | Sometimes |
| Age35-54 | -0.52 | 0.24 | -2.21 | **0.027** | Sometimes |
| Age [>=55] | -0.56 | 0.28 | -2.04 | **0.041** | Sometimes |
| Region [North] | 0.35 | 0.22 | 1.6 | 0.109 | Sometimes |
| Role [Nurse] | -0.35 | 0.23 | -1.57 | 0.117 | Sometimes |
| Unit [Anesthesia/Reanimation/Inten.care] | -0.41 | 0.42 | -0.96 | 0.337 | Sometimes |
| Unit [New COVID unit] | -0.19 | 0.26 | -0.73 | 0.467 | Sometimes |
|  |  |  |  |  |  |
| (Intercept) | -0.39 | 0.36 | -1.09 | 0.276 | Often |
| Gender [M] | -0.51 | 0.3 | -1.71 | 0.088 | Often |
| Age35-54 | -0.37 | 0.29 | -1.26 | 0.207 | Often |
| Age [>=55] | -1.07 | 0.39 | -2.72 | **0.007** | Often |
| Region [North] | 0.32 | 0.29 | 1.1 | 0.27 | Often |
| Role [Nurse] | -0.53 | 0.29 | -1.84 | 0.066 | Often |
| Unit [Anesthesia/Reanimation/Inten.care] | -0.59 | 0.6 | -0.99 | 0.321 | Often |
| Unit [New COVID unit] | -0.17 | 0.34 | -0.51 | 0.611 | Often |
|  |  |  |  |  |  |
| (Intercept) | -3.26 | 1.03 | -3.16 | **0.002** | Almost always or always |
| Gender [M] | -0.2 | 0.72 | -0.27 | 0.787 | Almost always or always |
| Age35-54 | -0.24 | 0.73 | -0.33 | 0.74 | Almost always or always |
| Age [>=55] | -0.73 | 0.96 | -0.77 | 0.444 | Almost always or always |
| Region [North] | 0.97 | 0.82 | 1.19 | 0.234 | Almost always or always |
| Role [Nurse] | -0.1 | 0.76 | -0.13 | 0.9 | Almost always or always |
| Unit [Anesthesia/Reanimation/Inten.care] | -12.26 | 432.15 | -0.03 | 0.977 | Almost always or always |
| Unit [New COVID unit] | -1 | 1.07 | -0.93 | 0.351 | Almost always or always |

*Significant P values are highlighted in bold.*

| **Supplementary table 16.** Models coefficients, standard errors and relative significance for Quiet | | | | | |
| --- | --- | --- | --- | --- | --- |
| **Quiet** | | | | | |
| **Predictors** | **Estimate** | **std. Error** | **z-Stat** | ***P*-value** | **Response** |
| (Intercept) | 0.75 | 0.33 | 2.3 | **0.021** | Sometimes |
| Gender [M] | -0.33 | 0.27 | -1.22 | 0.224 | Sometimes |
| Age35-54 | 0.21 | 0.27 | 0.79 | 0.427 | Sometimes |
| Age [>=55] | 0.21 | 0.34 | 0.62 | 0.534 | Sometimes |
| Region [North] | -0.02 | 0.26 | -0.09 | 0.932 | Sometimes |
| Role [Nurse] | 0.11 | 0.26 | 0.4 | 0.686 | Sometimes |
| Unit [Anesthesia/Reanimation/Inten.care] | 0.91 | 0.66 | 1.38 | 0.168 | Sometimes |
| Unit [New COVID unit] | -0.2 | 0.29 | -0.68 | 0.496 | Sometimes |
|  |  |  |  |  |  |
| (Intercept) | -0.46 | 0.38 | -1.21 | 0.226 | Often |
| Gender [M] | 0.46 | 0.28 | 1.64 | 0.102 | Often |
| Age35-54 | 0.52 | 0.31 | 1.67 | 0.095 | Often |
| Age [>=55] | 1.23 | 0.37 | 3.35 | **0.001** | Often |
| Region [North] | -0.12 | 0.29 | -0.42 | 0.677 | Often |
| Role [Nurse] | 0.42 | 0.3 | 1.41 | 0.159 | Often |
| Unit [Anesthesia/Reanimation/Inten.care] | 1.21 | 0.68 | 1.76 | 0.078 | Often |
| Unit [New COVID unit] | -0.53 | 0.35 | -1.52 | 0.128 | Often |
|  |  |  |  |  |  |
| (Intercept) | -3.5 | 0.84 | -4.18 | **<0.001** | Almost always or always |
| Gender [M] | 1.54 | 0.48 | 3.19 | **0.001** | Almost always or always |
| Age35-54 | 1.41 | 0.64 | 2.22 | **0.027** | Almost always or always |
| Age [>=55] | 1.33 | 0.77 | 1.73 | 0.084 | Almost always or always |
| Region [North] | -0.73 | 0.49 | -1.5 | 0.134 | Almost always or always |
| Role [Nurse] | 1.14 | 0.56 | 2.06 | **0.04** | Almost always or always |
| Unit [Anesthesia/Reanimation/Inten.care] | 1.61 | 1.01 | 1.59 | 0.112 | Almost always or always |
| Unit [New COVID unit] | -0.19 | 0.63 | -0.3 | 0.762 | Almost always or always |

*Significant P values are highlighted in bold.*

| **Supplementary table 17.** Models coefficients, standard errors and relative significance for Solidarity | | | | | |
| --- | --- | --- | --- | --- | --- |
| **Solidarity** | | | | | |
| **Predictors** | **Estimate** | **std. Error** | **z-Stat** | ***P*-value** | **Response** |
| (Intercept) | 1.37 | 0.57 | 2.42 | **0.015** | Sometimes |
| Gender [M] | -0.83 | 0.45 | -1.86 | 0.062 | Sometimes |
| Age35-54 | 0.13 | 0.45 | 0.29 | 0.77 | Sometimes |
| Age [>=55] | 1.44 | 0.84 | 1.71 | 0.088 | Sometimes |
| Region [North] | 0.41 | 0.45 | 0.91 | 0.362 | Sometimes |
| Role [Nurse] | -0.15 | 0.49 | -0.3 | 0.763 | Sometimes |
| Unit [Anesthesia/Reanimation/Inten.care] | 13.72 | 0.34 | 40.45 | **<0.001** | Sometimes |
| Unit [New COVID unit] | 0.15 | 0.62 | 0.24 | 0.81 | Sometimes |
|  |  |  |  |  |  |
| (Intercept) | 1.56 | 0.55 | 2.84 | **0.004** | Often |
| Gender [M] | -1.16 | 0.43 | -2.69 | **0.007** | Often |
| Age35-54 | 0.75 | 0.44 | 1.72 | 0.086 | Often |
| Age [>=55] | 2.3 | 0.82 | 2.8 | **0.005** | Often |
| Region [North] | 0.88 | 0.43 | 2.06 | **0.039** | Often |
| Role [Nurse] | -0.25 | 0.47 | -0.52 | 0.605 | Often |
| Unit [Anesthesia/Reanimation/Inten.care] | 13.46 | 0.32 | 42.46 | **<0.001** | Often |
| Unit [New COVID unit] | 0.26 | 0.59 | 0.44 | 0.657 | Often |
|  |  |  |  |  |  |
| (Intercept) | 0.53 | 0.6 | 0.88 | 0.379 | Almost always or always |
| Gender [M] | -0.77 | 0.46 | -1.68 | 0.093 | Almost always or always |
| Age35-54 | 0.58 | 0.48 | 1.21 | 0.227 | Almost always or always |
| Age [>=55] | 2.35 | 0.85 | 2.77 | **0.006** | Almost always or always |
| Region [North] | 0.82 | 0.46 | 1.78 | 0.075 | Almost always or always |
| Role [Nurse] | -0.27 | 0.51 | -0.53 | 0.594 | Almost always or always |
| Unit [Anesthesia/Reanimation/Inten.care] | 12.2 | 0.5 | 24.2 | **<0.001** | Almost always or always |
| Unit [New COVID unit] | 0.64 | 0.61 | 1.05 | 0.296 | Almost always or always |

*Significant P values are highlighted in bold.*

| **Supplementary table 18.** Models coefficients, standard errors and relative significance for Hope | | | | | |
| --- | --- | --- | --- | --- | --- |
| **Hope** | | | | | |
| **Predictors** | **Estimate** | **std. Error** | **z-Stat** | ***P*-value** | **Response** |
| (Intercept) | 1.2 | 0.43 | 2.82 | **0.005** | Sometimes |
| Gender [M] | -0.63 | 0.34 | -1.88 | 0.06 | Sometimes |
| Age35-54 | 0.25 | 0.35 | 0.7 | 0.484 | Sometimes |
| Age [>=55] | 0.6 | 0.49 | 1.22 | 0.221 | Sometimes |
| Region [North] | 0.21 | 0.34 | 0.61 | 0.543 | Sometimes |
| Role [Nurse] | 0.02 | 0.35 | 0.06 | 0.953 | Sometimes |
| Unit [Anesthesia/Reanimation/Inten.care] | 0.49 | 0.69 | 0.7 | 0.481 | Sometimes |
| Unit [New COVID unit] | -0.35 | 0.41 | -0.84 | 0.399 | Sometimes |
|  |  |  |  |  |  |
| (Intercept) | 0.79 | 0.43 | 1.83 | 0.067 | Often |
| Gender [M] | -0.67 | 0.33 | -2.01 | **0.044** | Often |
| Age35-54 | 0.69 | 0.35 | 1.98 | **0.048** | Often |
| Age [>=55] | 1.4 | 0.48 | 2.92 | **0.004** | Often |
| Region [North] | 0.31 | 0.34 | 0.91 | 0.363 | Often |
| Role [Nurse] | 0.28 | 0.35 | 0.81 | 0.418 | Often |
| Unit [Anesthesia/Reanimation/Inten.care] | 0.15 | 0.71 | 0.21 | 0.833 | Often |
| Unit [New COVID unit] | -0.17 | 0.4 | -0.41 | 0.679 | Often |
|  |  |  |  |  |  |
| (Intercept) | -0.92 | 0.58 | -1.59 | 0.112 | Almost always or always |
| Gender [M] | -0.28 | 0.4 | -0.7 | 0.483 | Almost always or always |
| Age35-54 | 1.32 | 0.49 | 2.7 | **0.007** | Almost always or always |
| Age [>=55] | 2.12 | 0.6 | 3.51 | **<0.001** | Almost always or always |
| Region [North] | -0.11 | 0.41 | -0.27 | 0.788 | Almost always or always |
| Role [Nurse] | 0.25 | 0.43 | 0.57 | 0.569 | Almost always or always |
| Unit [Anesthesia/Reanimation/Inten.care] | 0.4 | 0.89 | 0.45 | 0.652 | Almost always or always |
| Unit [New COVID unit] | 0.02 | 0.49 | 0.05 | 0.961 | Almost always or always |

*Significant P values are highlighted in bold.*

| **Supplementary table 19.** Models coefficients, standard errors and relative significance for Trust | | | | | |
| --- | --- | --- | --- | --- | --- |
| **Trust** | | | | | |
| **Predictors** | **Estimate** | **std. Error** | **z-Stat** | ***P*-value** | **Response** |
| (Intercept) | 1.03 | 0.38 | 2.7 | **0.007** | Sometimes |
| Gender [M] | 0.05 | 0.32 | 0.17 | 0.868 | Sometimes |
| Age35-54 | 0.62 | 0.31 | 1.97 | **0.049** | Sometimes |
| Age [>=55] | 0.58 | 0.4 | 1.45 | 0.148 | Sometimes |
| Region [North] | 0.05 | 0.31 | 0.17 | 0.868 | Sometimes |
| Role [Nurse] | -0.06 | 0.32 | -0.18 | 0.86 | Sometimes |
| Unit [Anesthesia/Reanimation/Inten.care] | 0.76 | 0.79 | 0.96 | 0.337 | Sometimes |
| Unit [New COVID unit] | -0.6 | 0.34 | -1.77 | 0.077 | Sometimes |
|  |  |  |  |  |  |
| (Intercept) | 0.3 | 0.41 | 0.74 | 0.462 | Often |
| Gender [M] | 0.34 | 0.33 | 1.02 | 0.308 | Often |
| Age35-54 | 1.03 | 0.33 | 3.09 | **0.002** | Often |
| Age [>=55] | 1.03 | 0.42 | 2.46 | **0.014** | Often |
| Region [North] | 0.11 | 0.33 | 0.32 | 0.747 | Often |
| Role [Nurse] | 0.03 | 0.33 | 0.08 | 0.94 | Often |
| Unit [Anesthesia/Reanimation/Inten.care] | 1.01 | 0.8 | 1.27 | 0.205 | Often |
| Unit [New COVID unit] | -0.74 | 0.36 | -2.05 | **0.041** | Often |
|  |  |  |  |  |  |
| (Intercept) | -2.48 | 0.76 | -3.26 | **0.001** | Almost always or always |
| Gender [M] | 1.1 | 0.47 | 2.33 | **0.020** | Almost always or always |
| Age35-54 | 1.63 | 0.63 | 2.57 | **0.010** | Almost always or always |
| Age [>=55] | 2.15 | 0.7 | 3.06 | **0.002** | Almost always or always |
| Region [North] | -0.39 | 0.48 | -0.81 | 0.415 | Almost always or always |
| Role [Nurse] | 0.51 | 0.52 | 0.99 | 0.323 | Almost always or always |
| Unit [Anesthesia/Reanimation/Inten.care] | 0.5 | 1.29 | 0.38 | 0.7 | Almost always or always |
| Unit [New COVID unit] | -0.32 | 0.56 | -0.57 | 0.569 | Almost always or always |

*Significant P values are highlighted in bold.*

| **Supplementary table 20.** Models coefficients, standard errors and relative significance for Satisfaction | | | | | |
| --- | --- | --- | --- | --- | --- |
| **Satisfaction** | | | | | |
| **Predictors** | **Estimate** | **std. Error** | **z-Stat** | ***P*-value** | **Response** |
| (Intercept) | 1.31 | 0.37 | 3.59 | **<0.001** | Sometimes |
| Gender [M] | -0.73 | 0.28 | -2.62 | **0.009** | Sometimes |
| Age35-54 | 0.25 | 0.29 | 0.89 | 0.374 | Sometimes |
| Age [>=55] | 0.5 | 0.36 | 1.39 | 0.165 | Sometimes |
| Region [North] | 0.07 | 0.28 | 0.25 | 0.803 | Sometimes |
| Role [Nurse] | -0.58 | 0.3 | -1.97 | **0.049** | Sometimes |
| Unit [Anesthesia/Reanimation/Inten.care] | 0.51 | 0.61 | 0.83 | 0.405 | Sometimes |
| Unit [New COVID unit] | 0.3 | 0.36 | 0.82 | 0.41 | Sometimes |
|  |  |  |  |  |  |
| (Intercept) | 0.36 | 0.4 | 0.9 | 0.367 | Often |
| Gender [M] | -0.31 | 0.29 | -1.07 | 0.285 | Often |
| Age35-54 | 0.64 | 0.31 | 2.07 | **0.038** | Often |
| Age [>=55] | 0.87 | 0.39 | 2.26 | **0.024** | Often |
| Region [North] | -0.18 | 0.29 | -0.62 | 0.536 | Often |
| Role [Nurse] | -0.03 | 0.31 | -0.08 | 0.934 | Often |
| Unit [Anesthesia/Reanimation/Inten.care] | 0.84 | 0.62 | 1.35 | 0.177 | Often |
| Unit [New COVID unit] | 0.36 | 0.38 | 0.96 | 0.339 | Often |
|  |  |  |  |  |  |
| (Intercept) | -2.72 | 0.82 | -3.32 | **0.001** | Almost always or always |
| Gender [M] | 0.75 | 0.49 | 1.53 | 0.126 | Almost always or always |
| Age35-54 | 1.19 | 0.63 | 1.88 | 0.06 | Almost always or always |
| Age [>=55] | 1.4 | 0.75 | 1.87 | 0.062 | Almost always or always |
| Region [North] | -0.59 | 0.51 | -1.17 | 0.24 | Almost always or always |
| Role [Nurse] | 0.44 | 0.56 | 0.78 | 0.437 | Almost always or always |
| Unit [Anesthesia/Reanimation/Inten.care] | 0.65 | 1.2 | 0.54 | 0.589 | Almost always or always |
| Unit [New COVID unit] | 1.14 | 0.58 | 1.97 | **0.049** | Almost always or always |

*Significant P values are highlighted in bold.*

| **Supplementary table 21.** Models coefficients, standard errors and relative significance for Pride | | | | | |
| --- | --- | --- | --- | --- | --- |
| **Pride** | | | | | |
| **Predictors** | **Estimate** | **std. Error** | **z-Stat** | ***P*-value** | **Response** |
| (Intercept) | -0.09 | 0.3 | -0.29 | 0.774 | Sometimes |
| Gender [M] | 0.16 | 0.24 | 0.67 | 0.502 | Sometimes |
| Age35-54 | 0.12 | 0.25 | 0.48 | 0.63 | Sometimes |
| Age [>=55] | -0.2 | 0.29 | -0.67 | 0.503 | Sometimes |
| Region [North] | 0 | 0.24 | 0 | 0.997 | Sometimes |
| Role [Nurse] | 0.28 | 0.24 | 1.16 | 0.248 | Sometimes |
| Unit [Anesthesia/Reanimation/Inten.care] | 0.33 | 0.46 | 0.72 | 0.474 | Sometimes |
| Unit [New COVID unit] | 0.25 | 0.3 | 0.85 | 0.398 | Sometimes |
|  |  |  |  |  |  |
| (Intercept) | -0.69 | 0.34 | -2 | **0.045** | Often |
| Gender [M] | 0.43 | 0.26 | 1.68 | 0.092 | Often |
| Age35-54 | 0.29 | 0.28 | 1.05 | 0.296 | Often |
| Age [>=55] | -0.03 | 0.33 | -0.08 | 0.936 | Often |
| Region [North] | -0.06 | 0.26 | -0.23 | 0.818 | Often |
| Role [Nurse] | 0.3 | 0.27 | 1.11 | 0.267 | Often |
| Unit [Anesthesia/Reanimation/Inten.care] | 0.32 | 0.51 | 0.63 | 0.528 | Often |
| Unit [New COVID unit] | 0.42 | 0.32 | 1.32 | 0.186 | Often |
|  |  |  |  |  |  |
| (Intercept) | -2.44 | 0.63 | -3.88 | **<0.001** | Almost always or always |
| Gender [M] | 0.2 | 0.43 | 0.46 | 0.643 | Almost always or always |
| Age35-54 | 0.93 | 0.54 | 1.73 | 0.084 | Almost always or always |
| Age [>=55] | 0.75 | 0.6 | 1.25 | 0.212 | Almost always or always |
| Region [North] | -0.22 | 0.42 | -0.53 | 0.598 | Almost always or always |
| Role [Nurse] | 0.28 | 0.44 | 0.62 | 0.533 | Almost always or always |
| Unit [Anesthesia/Reanimation/Inten.care] | -12.15 | 390.97 | -0.03 | 0.975 | Almost always or always |
| Unit [New COVID unit] | 0.66 | 0.48 | 1.38 | 0.168 | Almost always or always |

*Significant P values are highlighted in bold.*
